# Sex Differences in Gut Microbial Development of Preterm Infant Twins in Early Life: A Longitudinal Analysis

**DOI:** 10.3389/fcimb.2021.671074

**Published:** 2021-08-12

**Authors:** Jie Chen, Hongfei Li, Sarah M. Hird, Ming-Hui Chen, Wanli Xu, Kendra Maas, Xiaomei Cong

**Affiliations:** ^1^School of Nursing, University of Connecticut, Storrs, CT, United States; ^2^Department of Statistics, University of Connecticut, Storrs, CT, United States; ^3^Department of Molecular and Cell Biology, University of Connecticut, Storrs, CT, United States; ^4^Microbial Analysis, Resources, and Services (MARS), University of Connecticut, Storrs, CT, United States; ^5^Institute for Systems Genomics, University of Connecticut, Farmington, CT, United States

**Keywords:** preterm infants, twins and triplets, sex, neonatal intensive care unit, gut microbiota

## Abstract

Infant gut microbiota plays a vital role in immune response, mediates neurobehavioral development and health maintenance. Studies of twins’ gut microbiota found that gut microbiota composition and diversity tend to be mature and stable with increasing postnatal age (PNA). Preterm infant gut microbiome shifts dramatically when they were staying in the neonatal intensive care unit (NICU). Compositions and shifting characteristics of gut microbiota among neonatal preterm twins and triplets during their early life are still unknown, which impedes a better understanding of the mechanism underpinning neurobehavioral development and precise intervention/health of preterm neonates. This longitudinal cohort study used a twins/triplets design to investigate the interaction of genetic (e.g., male *vs.* female) and environmental factors influencing the development of the gut microbiome in early life. We included 39 preterm infants, 12 were Female twins/triplets (Female T/T) including 3 twins pairs and 2 triplets, 12 were male twins (Male T) including 6 twins pairs, and 15 were mixed-sex twins/triplets (Mix T/T) including 6 twins pairs and 1 triplet (8 females and 7 males) during the first four weeks of NICU stay. Weekly gut microbiota patterns between females and males were compared by linear discriminant analysis (LDA) effect size (LEfSe). Metagenomics function of gut microbiota was predicted by using Phylogenetic Investigation of Communities by Reconstruction of Unobserved States (PICRUSt). Weekly function (KEGG pathways) differences between females and males were detected by using Statistical Analysis of Metagenomic Profiles (STAMP). Results found that female pairs and male pairs were significantly different in gut microbiome diversity, compositions, and predicted metabolic profiles, importantly, females and males were also significantly dissimilar within their co-twin/triplet pairs of the mixed-sex group, infants of co-twins/triplets shared more similar features than un-related infants from different twins’ pair. Future research developing personalized interventions for vulnerable high-risk infants should consider sex, and the interaction of sex and environmental factors.

## Introduction

The microorganisms living in the gut are crucial to human health and provide essential benefits to its host, including dietary energy extraction, development and maintenance of the immune system, and protection against pathogens (Baumann-Dudenhoeffer et al., 2018; Dzidic et al., 2018; Adak and Khan, 2019; Parker et al., 2020). In the early stage of human life, the development of the gut microbiome is one of the most important bases for health and health-related issues potentially evolve throughout the life span (Cong et al., 2016b; Soderborg et al., 2018; Wandro et al., 2018; Selma-Royo et al., 2020; Shanahan et al., 2021). Multiple and complex factors influence the dynamics of the gut microbiome in early life, including age, sex, delivery mode, feeding, medication use, and the environment of hospitalization (Hill et al., 2017; Ramani et al., 2018; Wampach et al., 2018; Hou et al., 2020). When infants are born at high risk, i.e, prematurely, the colonization of gut microbiota is even more challenged and complicated due to the host developmental immaturity, difficulties of breastfeeding initiation, early life stress, and sophisticated medical environments for survival (Cong et al., 2016b). While the dysbiosis or imbalance of the microbial community is linked to a number of health changes in early life, the underlying mechanisms and temporal development of the microbiota remain largely understudied.

An interesting observation in the neonatal intensive care unit (NICU), as well as research evidence, show that premature female infants are more likely to thrive and have better health outcomes compared to their male counterparts (Zisk et al., 2011; Van Nostrand et al., 2015; Shim et al., 2017). Likewise, male preterm infants are more vulnerable to major morbidities such as respiratory and neurological dysfunctions, in spite of their heavier body weight distribution than females at birth (Zhao et al., 2017). The sex benefit of females or the disadvantage of males in clinical outcomes is multifactorial and may be regulated by the interplay between hormonal, immunological, and genetic factors. Importantly, these factors may affect the sex differences in the development of the gut microbiome, and in turn, sex predispositions of certain gut microbiome patterns and functions contribute to differential health outcomes among male and female infants (Levy et al., 2015; Negi et al., 2019; Zeevi et al., 2019). The current knowledge of sex differences in preterm infants is still limited, particularly, how sex-specific microbial colonization influence preterm health is largely unknown.

Compared to full-term neonates, the gut microbiome community of preterm infants often present with reduced levels of obligate anaerobes and increased abundances of facultative anaerobe, such as *Enterobacter*, *Enterococcus*, *Escherichia*, and *Klebsiella* (Cong et al., 2016a; Henderickx et al., 2019; Younge et al., 2019). When compared to the female preterm infants, males were found to have a lower α-diversity of the gut microbiome after birth, as well, males were more likely to have a higher abundance of *Enterobacteriales* and lower abundance of *Clostridiates* than females in early life (Cong et al., 2017). Sex differences in the gut microbiome composition after birth may contribute to the sex-specific health risk or diseases across the lifespan, including mortality and morbidities in infancy as well as autoimmune and metabolic disorders in later life (Jašarević et al., 2016).

In the present study, we used a twin study design to investigate the interaction of sex (e.g., male vs. female) and environmental factors influencing the development of the gut microbiome in early life. Due to their unique genetic attributes, twins/triplets play a valuable role in understanding the complex mechanisms of the highly dynamic microbiome along with the development of the infant. One twin study found that the virome and bacterial microbiome were more similar between co-twins than between non-related infants from birth to two years of age, demonstrating that co-twin infants shared a more similar bacterial microbiome compared to unrelated individuals (Lim et al., 2015). Nevertheless, the dynamic evolution of gut microbiota in preterm infants, the interplay of genetic factors, e.g., sex and environment on the microbiome, as well as the impact of gut microbiota on infant health and development remain unclear.

Given that preterm infants’ health status and their gut microbiota shift when they stay in NICU and these dynamic changes may have long-term effects on their health, the purpose of this study was to explore the developmental patterns of the gut microbiome in preterm twins/triplets during their first 4 weeks of stay in the NICU. Meanwhile, the study aimed to investigate the sex differences in gut microbiome development over time in co-twin/triplet pairs and non-related infants, including uni-sex twins/triplets (e.g., female pairs and male pairs) and mixed-sex twins/triplets of preterm infants.

## Materials and Methods

### Study Design and Participants

A prospective exploratory study was conducted at level IV NICUs at two sites in the Northeastern United States and a twin study design was used in this analysis. The twin study design allowed us to control the genetic variations within each twin/triplet pairs. The maternal factors and the environmental conditions were also controlled because the twins stayed in the same NICU and received similar treatment regimens provided by the same healthcare team. Preterm twins/triplets included in this report were a subset of samples recruited from a study funded by the National Institutes of Health (NIH). Stable preterm infants were recruited when they were admitted to the NICUs from 2013 – 2016. The *Inclusion criteria* were: 1) 28 0/7 – 32 6/7 weeks gestational age, 2) 0 - 7 days old after birth, and 3) infant mothers were older than 18 years old to provide consent. *Exclusion criteria* were infants who had: 1) known congenital anomalies, 2) severe periventricular/intraventricular hemorrhage (≥ Grade III), 3) undergone minor or major surgery procedures, and/or 4) positive drug exposure history. In this particular study, only multiple-birth infants, either monozygotic or dizygotic twins/triplets were included in the analysis. All study protocols were performed in accordance with relevant guidelines and regulations and approved by the institutional review boards (IRBs) of the study hospital and the affiliated university. Written informed consent was obtained from the parents of all twins/triplets. After being enrolled in the study, preterm infants were followed up over their first 28 days of life in the NICU.

### Measurements and Data Collection

Demographic information and infant health characteristics were abstracted by the research staffs from the medical record. The Score for Neonatal Acute Physiology – Perinatal Extension-II (SNAPPE-II) (Richardson et al., 2001) was used to measure the severity of the illness of the infant. Data of antibiotic administration were also collected, such as days and doses of antibiotic use during the NICU hospitalization. Feeding regiments of these infants were collected by the research nurses at the NICUs, including the frequency of infant fed by mother’s own breastmilk, human donor milk, and/or formula over the first 28 days of life.

Infant fecal samples were collected by trained nurses at NICUs on a daily basis, depending upon whether the infant had a stool. Samples were immediately frozen upon collection at -80°C, then transferred on dry ice to the laboratory and stored at -80°C until processing. All stool samples were assigned a unique study ID number and systematically entered into a specimen repository and database.

### Stool Sample DNA Extraction, Sequencing, and Data Processing

The DNA extraction, extraction, and processing method and procedures were used as described previously (Cong et al., 2016a; Cong et al., 2017). DNA was extracted from 0.25g of a fecal sample using the MoBio Power Soil kit (MoBio Laboratories, Inc) according to the manufacturer’s instruction for the Eppendorf epMotion 5075 Vac liquid handling robot or manually. DNA extracts were quantified using a Synergy HT (Biotek, Winooski, VT) with the Quant-iT PicoGreen kit (Invitrogen, ThermoFisher Scientific). The V4 regions of the 16S rRNA gene were amplified with 515F and 806R primers containing Illumina adapters and golay indices on the 3’ end using 20 ng extracted DNA as a template. Samples were amplified in triplicate using Phusion High-Fidelity PCR master mix (New England BioLabs) with the addition of 10 μg BSA (New England BioLabs). The PCR reaction was incubated at 95°C for 3.5 minutes, the 30 cycles of 30 s at 95.0°C, 30 s at 50.0°C, and 90 s at 72.0°C, followed by a final extension at 72.0°C for 10 minutes. PCR products were quantified and visualized using the QIAxcel DNA Fast Analysis (Qiagen). PCR products were normalized based on the concentration of DNA in the 350–400 bp region and pooled using the QIAgility liquid handling robot (Qiagen). Pooled PCR products were cleaned using the Gene Read Size Selection kit (Qiagen) according to the manufacturer’s protocol. The cleaned pool was sequenced on the MiSeq using a v2 2x250 base-pair kit (Illumina, Inc). Using Mothur 1.42.3 pipeline (Schloss et al., 2009), operational taxonomic units (OTUs) were determined by clustering reads to the Greengenes reference 16S reference dataset (January 2018 release) at a 97% identity, and then performing *de novo* OTU clustering on reads that failed to cluster to a reference. Taxonomic annotation was also determined by the Greengenes reference 16S reference dataset.

### Statistical Analysis

Clinical data, OTU tables, α-diversity, and β-diversity index calculated from Mothur process were imported into R 3.6.2 for statistical analysis. The Shannon index, and species of observation (SOBS), were calculated to measure the α-diversity index. The Bray-Curtis, Jaccard, and Theta YC dissimilarities index were calculated to present β-diversity of the Bacterial Community.

Linear mixed effect models were used to model and make inferences for the longitudinal a-diversity data. Levene’s Test for Homogeneity of Variance was used to determine whether homoscedasticity or heteroscedasticity assumptions should be applied. To make inference on how different factors influence richness index (SOBS) and evenness index (Shannon diversity) of the Gut Microbiota, demographics variables including delivery type, birth weight, birth length, twin types (female, male vs mixed), premature rupture of membranes (PROM) or not, baseline Apgar score at 1 min, and baseline SNAPPE-II score were cooperated into the model as among-individual covariates; PNA, the total number of mother’s breast milk feeding in 3 days before each sample were considered as within-individual covariates; interactions between twin types and PNA were also included. Individual-specific intercept and slope were included as random components in the models, and the variations for each of the 3 twin-types groups were allowed to be different.

For β-diversity indexes, comparisons among three twin types groups across averaged PNA (day) were performed using Kruskal–Wallis test. Then, the comparison between co-twins/triples compared to non-related infants from both *same sex* and *different sex* were made *via* the Mann-Whitney U test. Box plots were used to illustrate the population trend of the β-diversity index for different twin types across co-twins pairs and non-related infants pairs. The diversity measures are logit-transformed in the box-plot to provide clear information on the tails.

Exploratory data analyses including taxonomy graph techniques were conducted to display the composition of the organism in the preterm infants’ gut-microbiome community. First, weekly stacked bar plots for selected Taxonomic for all twins/triplets presented the overall compositional pattern and the longitudinally feature of our data. Spaghetti plots for α-diversity index against baby’s PNA displayed the dynamics in the early life for preterm babies. Weekly differences of gut microbiota composition between female twins/triplets and male twins, and the difference between female infants and male infants among mixed twins/triplets were also compared by LEfSe (Segata et al., 2011). The alpha value for the factorial Kruskal–Wallis test was set to 0.05 as well as for the pairwise Wilcoxon test, and the threshold on the logarithmic LDA score for discriminative features was set as 2.0. The results were visualized in the form of LDA score and taxonomic cladograms.

Metagenomics functions of different gut microbiota compositions were predicted by using Phylogenetic Investigation of Communities by Reconstruction of Unobserved States (PICRUSt) (Langille et al., 2013). Weekly function (KEGG pathways) differences between the two sexes were detected by using Statistical Analysis of Metagenomic Profiles (STAMP) (Parks and Beiko, 2010). For the pathway analysis, the Kruskal-Wallis test was used to compare the difference between Female T/T and Male T, and also the difference between females and males among Mixed T/T. Benjamini-Hochberg procedure was used to decreases the false discovery rate for multiple test corrections. The p-value was set as 0.05.

## Results

### Characterization of the Preterm Twins and Triplets

A total of 41 preterm infants (16 twins and 3 triplets) were enrolled in this study. One-paired twins were not included in the analysis due to the low quality of the stool sample sequence. In the final analysis of 39 subjects, 12 were females of twins/triplets (Female T/T; 3 twins pairs and 2 triplets), 12 were male twins (Male T; 6 pairs), and 15 were mixed-sex twins/triplets (Mixed T/T; 6 twins pairs and 1 triplet; 8 females and 7 males). Seven Female T/T, seven Mixed T/T, and all the Male T were from one NICU, the other five Female T/T and eight Mixed T/T were from the other NICU (p-value = 0.006), but both NICUs have been managed and served by the same team of health providers. Demographic and clinical characterizations of these infants were summarized in **Table 1**.

**Table 1:** Demographic information and clinical characteristics of preterm twins/triplets (n=39).

	Female T/T (n = 12)	Male T (n = 12)	Mixed T/T (n = 15)	p-value
Gestational age at birth (weeks) [median (IQR)]	30.6 (2.0)	32.4 (1.1)	30.6 (2.1)	0.0425
C-cession (%)	10 (83.3)	6 (50.0)	13 (86.7)	0.066
Pre-mature rupture of membrane (%)	4 (33.3)	7 (58.3)	11 (73.3)	0.113
Resuscitation (%)	9 (75.0)	10 (83.3)	14 (93.3)	0.418
Race (%)				0.586
White	10 (83.3)	8 (66.7)	12 (80.0)	
Non-white	2 (16.7)	4 (33.3)	3 (20.0)	
American African	0	4 (33.3)	3 (20.0)	
2 or more	2 (16.7)	0	0	
Height (cm) [median (IQR)]	40.5 (5.3)	41.2 (4.3)	41 (3.5)	0.552
Weight (kg) [median (IQR)]	1.238 (0.594)	1.702 (0.392)	1.540 (0.405)	0.120
Head circumference (cm) [median (IQR)]	27.2 (3.0)	29.0 (1.3)	29.5 (3.8)	0.126
Apgar at 1 min [median (IQR)]	6 (2.5)	8 (1.3)	7 (3.0)	0.207
Apgar at 5 min [median (IQR)]	8 (1.0)	9 (1.0)	8 (1.0)	0.458
SNAPPE-II [median (IQR)]	9 (11.5)	0 (6.0)	9 (7.8)	0.387

SNAPPE-II, Score for Neonatal Acute Physiology with Perinatal Extension-II.

Feeding regimens of the study twins/triplets during the first 4 weeks of NICU stay are presented in **Table 2** and **Supplementary Figure 1**. The Wilks’ Lambda Test was used to compare the multivariate feeding regimens across three groups for each week. Female T/T tend to receive fewer times of mom’s breast milk per day (Mean = 1.5, SD = 2.7) than Male T (Mean = 4.0, SD = 3.3) and Mixed T/T (Mean = 3.2, SD = 3.4) in the first week of NICU stay (p-value = 0.0076). There was no significant difference in feeding types in the 2^nd^, 3^rd,^ and 4^th^ week among the three twins/triplets groups (p = 0.1645, p=0.2862, p=0.3776 for 2^nd^, 3^rd,^ and 4^th^ weeks respectively). The majority of infants received at least one dose of antibiotic in the first three days of NICU stay (Mean = 4.1, SD =3.6). Ten infants did not receive any antibiotic at all, including 1 triplet and 2 infants from Female T/T, 2 Male T pairs, and 1 infant from Mixed twins. After the first three days of NICU stay, two infants from Female T/T and 4 infants (2 female and 2 male) in Mixed T/T (one mixed-sex triplets and one male infant) received antibiotic doses at least one time. There was no significant difference in antibiotic use after day 3 between female babies and male babies based on Fisher’s exact test (p = 0.6614). The daily antibiotic dose of each infant was summarized in **Supplementary Table 1**.

**Table 2:** Feeding count of preterm twins/triplets (n=39) [mean (SD)].

Week	Feeding type	Female T/T (n = 12)	Male T (n = 12)	Mixed T/T (n = 15)	P value
1^st^	MBM	1.5 (2.7)	4.0 (3.3)	3.2 (3.4)	0.0076
	DBM	2.7 (3.4)	1.5 (2.5)	0.6 (1.8)	
	Formula	1.1 (2.7)	0.3 (1.3)	0.7 (1.8)	
2^nd^	MBM	4.3 (3.6)	4.4 (3.3)	5.5 (3.2)	0.1645
	DBM	2.4 (3.3)	2.1 (3.1)	0.8 (1.9)	
	Formula	1.3 (3.0)	0.9 (2.1)	0.3 (0.8)	
3^rd^	MBM	4.2 (3.7)	4.3 (3.3)	5.9 (2.9)	0.2862
	DBM	1.8 (2.7)	1.3 (2.9)	1.3 (2.3)	
	Formula	1.8 (2.9)	1.9 (2.5)	0.1 (0.4)	
4^th^	MBM	4.9 (3.5)	3.0 (3.2)	4.8 (3.3)	0.3776
	DBM	0.6 (1.4)	1.3 (2.8)	1.0 (1.9)	
	Formula	1.7 (3.0)	1.7 (2.5)	0.3 (1.1)	

MBM, mother’s breast milk; DBM, donor’s breast milk; Wilk’s Lambda test was performed.

### Sequencing of the Stool Samples

A total of 370 stool samples were collected and analyzed in this study. The average number of stool collections for each infant was 9.49 ± 3.96 with a median of 9 and a minimum of 2. Detailed information on stool sample collection of each infant was described in **Supplementary Table 2**. After sequencing, 370 fecal samples yielded 33,390,223 quality-filtered reads with an average of 90,243 reads per sample. In total, 5967 OTUs were identified and classified into 36 phyla, 104 classes, and 176 orders. *Proteobacteria, Firmicutes*, and *Bacteroidetes* were the most abundant phyla of the total reads. The microbiota data analysis was summarized in **Supplementary Figure 2**.

### The α-Diversity of Gut Microbiota Among the Preterm Twins and Triplets

**Figure 1:**
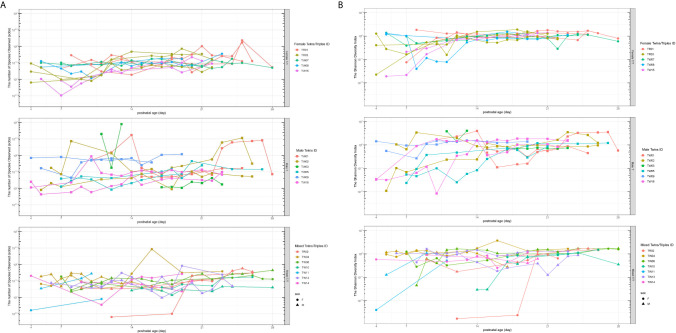
Richness (Sobs, **A**) and evenness (the Shannon diversity, **B**) of the bacterial community among twins/triplets over time. The subplots from top to bottom are for Female T/T, Male T, and Mixed T/T groups respectively. Each line in the spaghetti plot represents one baby and the same color in each subplot represents babies from the same mother. Sobs, species of observed.

The alpha diversity of the gut microbiota was measured using the species of observed (Sobs) and the Shannon diversity indices in this study, which provide quantitative measures of the abundance and evenness of the species in a microbiome community correspondingly. The Sobs (richness measure) and Shannon diversity (diversity measure) of all the three groups of preterm twins/triplets increased over the first 28 days (**Figures 1A, B**). Female T/T had significantly less inter-individual variation in both Sobs and Shannon diversity compared to Male T and Mixed T/T. The Levene’s Test for Homogeneity of Variance also indicates that three types of twins/triplets have different inter-personal variations (Sobs: p = 0.008, Shannon: p = 0.027).

Considering that several samples were missing from the data set, we also analyzed the diversity and its correlation to the parameters listed in **Table 3** using linear mixed effect models to discover the factors associated with the Sobs and Shannon diversity indices. The feature of heteroscedasticity of variations was allowed in the linear mixed effect models to account for different inter-individual variations. The results are shown in **Table 3**. For the richness of the bacterial community (Sobs), the infant’s postnatal age (PNA) was significantly associated with the Sobs of microbiota (p = 0.0011), which confirmed the increasing trend in **Figure 1A**. The APGAR-1min score, SNAPPE-II, and mother’s breast milk within 3 days of the gut samples collection seemed associated with the richness index, but did not reach the statistical significance. No difference of the Sobs was observed between different twin-type groups on the intercept and increasing trend in the model. For evenness of the bacterial community (Shannon Index), the infant’s PNA was significantly associated with the Shannon diversity (p = 0.0401), which was consistent with the overall increasing pattern of Shannon diversity as shown in **Figure 1B**. Apgar score at 1min was positively associated with the Shannon diversity (p = 0.0050), meanwhile, mother’s breast milk intake within 3 days prior to the gut microbiome samples was also significantly associated with increased Shannon diversity (p = 0.0002). Infant body length at birth, SNAPPE-II score, and PROM were correlated with the Shannon diversity index, but without statistical significance. No significant difference in the Shannon index was found across the three twin types.

**Table 3:** Factors associated with the Sobs and Shannon diversity indices, results from a linear mixed effect model.

	The Shannon Diversity			The number of species observed (Sobs)		
	Scaled B-coefficient	95% CI	p-value	Scaled B-coefficient	95% CI	p-value
Intercept	-4.693	(-9.726, 0.340)	0.0733	1.489	(-2.913, 5.891)	0.5150
Twinstype, Male T [ref. Female T/T]	-1.069	(-3.105, 0.966)	0.3001	0.497	(-0.501, 1.495)	0.3256
Twinstype, Mixed T/T [ref. Female T/T]	-1.101	(-3.149, 0.947)	0.2890	-0.057	(-1.067, 0.954)	0.9110
Postnatal age (day) * Twinstype [ref. Female T/T]	0.065	(0.004, 0.126)	**0.0401**	0.048	(0.02, 0.076)	**0.0011**
Postnatal age * Twinstype, Male T [ref. Female T/T]	0.040	(-0.046, 0.126)	0.3724	-0.019	(-0.062, 0.023)	0.3859
Postnatal age * Twinstype, Mixed T/T [ref. Female T/T]	0.012	(-0.074, 0.098)	0.7846	-0.006	(-0.047, 0.033)	0.7460
Delivery, C-section [ref. Vaginal]	0.102	(-0.602, 0.807)	0.7721	-0.001	(-0.592, 0.590)	0.9966
Premature rupture of membranes (PROM)	-0.512	(-1.146, 0.122)	0.1160	-0.057	(-0.619, 0.484)	0.8377
Body weight at birth	0.000	(-0.001, 0.001)	0.7584	0.000	(-0.001, 0.001)	0.9424
Body length at birth	0.084	(-0.045, 0.213)	0.2023	0.011	(-0.111, 0.133)	0.8589
Apgar score at birth (1 min)	0.152	(0.052, 0.252)	**0.0050**	0.057	(-0.035, 0.151)	0.2217
Apgar score at birth (5 min)	-0.041	(-0.289,0.208)	0.7432	0.031	(-0.192, 0.256)	0.7783
SNAPPE-II score at birth	0.013	(-0.014, 0.040)	0.3393	0.018	(-0.008, 0.044)	0.1713
Mother’s breast milk	0.029	(0.014, 0.044)	**0.0002**	0.004	(-0.009, 0.016)	0.5572

SNAPPE-II, Score for Neonatal Acute Physiology with Perinatal Extension-II. The symbol * refers to the interaction between two factors. The bold values mean the p- value was less than 0.05.

### The β-Diversity of the Preterm Twins and Triplets

The Bray-Curtis, Jaccard, and Theta YC dissimilarities index are the commonly used β-diversity indices to measure pairwise dissimilarity between the two microbiome samples, thus were used to present β-diversity of the gut microbiota community in this study. To obtain reliable and comparable results, we only included the pairs of microbiome samples from two babies when the corresponding postnatal age difference between two samples was within 7 days, and defined those pairs as a valid pair. For each valid pair of microbiome samples, their averaged postnatal age was calculated as the time index for the β-diversity measures. Boxplot was used at each averaged postnatal age to compare co-twins/triples with non-related infants. The comparisons and corresponding p-values were calculated for each averaged postnatal age between co-twins/triples and non-related infants by the Mann-Whitney test. P-values were presented on the graphs, and the significance found was colored in red for the figures in this section. The number of valid pairs of the same sex or different sex at each averaged postnatal age for co-twins and non-related infants across and among three twin types are presented in **Supplementary Table 3**. The pairs with averaged postnatal age before the 7^th^ postnatal day and after the 23^rd^ postnatal day were left out since the number of valid pairs of co-twins in any twin types groups was less than 10, which may present greater variabilities and produce unreliable trends and results. Thus, *the trend of β-diversity across three twin types in the second and third week of the babies’ NICU stay* and *the comparison between co-twins/triples with non-related infants* were our primary focus.

#### Overall Trend of β-Diversity Across Three Twin Types

**Figure 2:**
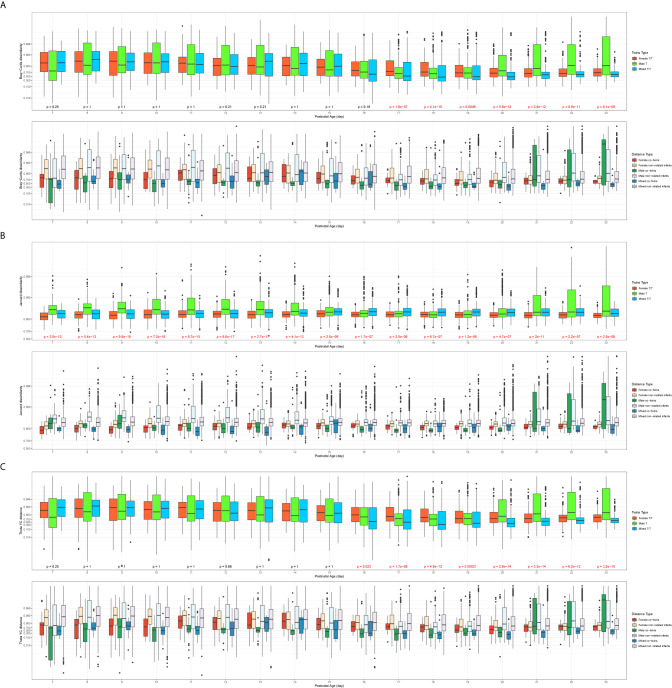
Overall trend of β-diversities across twin types (**A**: Bray-Curtis dissimilarity; **B**: Jaccard dissimilarity; **C**: Theta YC distance). In each of the subfigures, top panel: overall trend of β-diversities compared by the three twin types (Kruskal–Wallis test, significant p values colored in red); bottom panel: the trend of β-diversity across three twin types with co-twin pairs and non-related infants’ pairs; x-axis: the averaged postnatal age for sample pairs; y-axis: the logit-transformed β-diversity measures between 0 and 1.

Each type of β-diversity measures was calculated for each of three twin types communities and plotted over averaged postnatal age. **Figure 2** shows the dissimilarity indices in three twin types communities against the averaged postnatal age (day), including the overall trends across twin types (**Figure 2** top panels) and across twin types with co-twin pairs and non-related pairs (**Figure 2** bottom panels). The Male T communities had a larger Jaccard dissimilarity index than the Female T/T and Mixed T/T group up to 2 weeks, this trend was not clear after 2 weeks, but it was consistent among three β-diversity dissimilarity indices. **Figure 2** also shows significance among three twin types for all three β-diversity dissimilarities indices from day 17 to 23. Also, the sample pairs of each twin type can be further split into co-twins pairs or non-related infants’ pairs for all three β-diversity dissimilarities indices. The trend and relationship among six pair categories were also shown in **Figure 2**. For the non-related infants of the same sex, there was no clear trend between females and males. For the co-twins infants’ pairs of the same sex, males co-twins seem to have consistently lower β-diversity scores than females from day 7 to day 20, however, the trend doesn’t reach significance due to limited sample sizes. The comparison between co-twins/triples with non-related infants from the same sex or from different sex were presented in the following two sections.

#### Comparison of the β-Diversity of the Co-Twins/Triples With Non-Related Infants From the Same Sex

**Figure 3:**
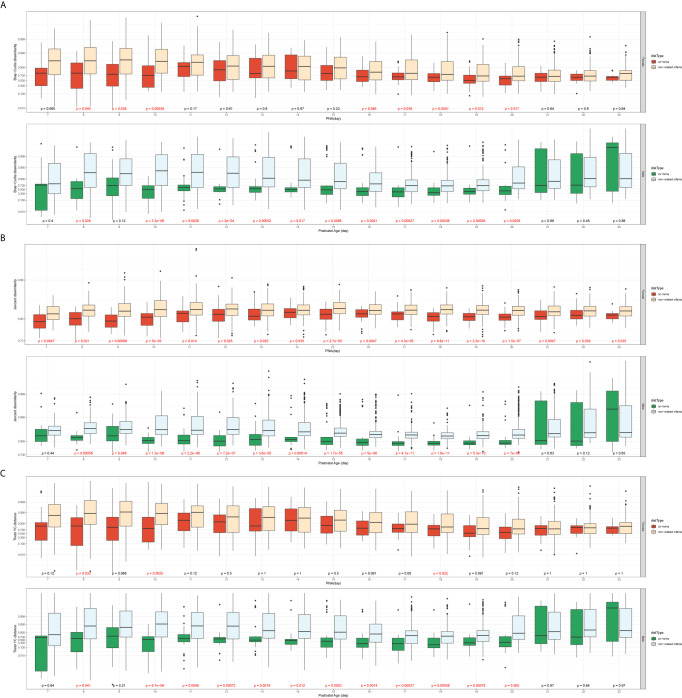
Comparison of the β-diversity of the co-twins/triples with non-related infants **from the same sex** (**A**: Bray-Curtis dissimilarity; **B**: Jaccard dissimilarity; **C**: Theta YC distance). In each of the subfigures, top panel: Female T/T (Mann-Whitney U test, significant p values colored in red); bottom panel: Male T (Mann-Whitney U test, significant p values colored in red); x-axis: the averaged postnatal age for sample pairs; y-axis: the logit-transformed β-diversity measures between 0 and 1.

The Female T/T group and Male T group were evaluated separately to avoid confounding between sex and twin types. **Figure 3** presents the dissimilarity index for sample pairs of the *same sex* against their averaged PNA. For all three β-diversity measures, the dissimilarities between co-twins/triples were less than those between non-related infants. For the Bray-Curtis and Theta YC dissimilarities index, the Male T group presented a statistically significant difference between co-twins/triples and non-related infants from day 10 to 20. In contrast, the Female T/T group presented little significance between co-twins/triples and non-related infants. For the Jaccard dissimilarities index, both Male T and Female T/T groups show a statistically significant trend that co-twins/triples were more similar than non-related infants.

#### Comparison of the Co-Twins/Triples With Non-Related Infants From the Different Sex

**Figure 4:**
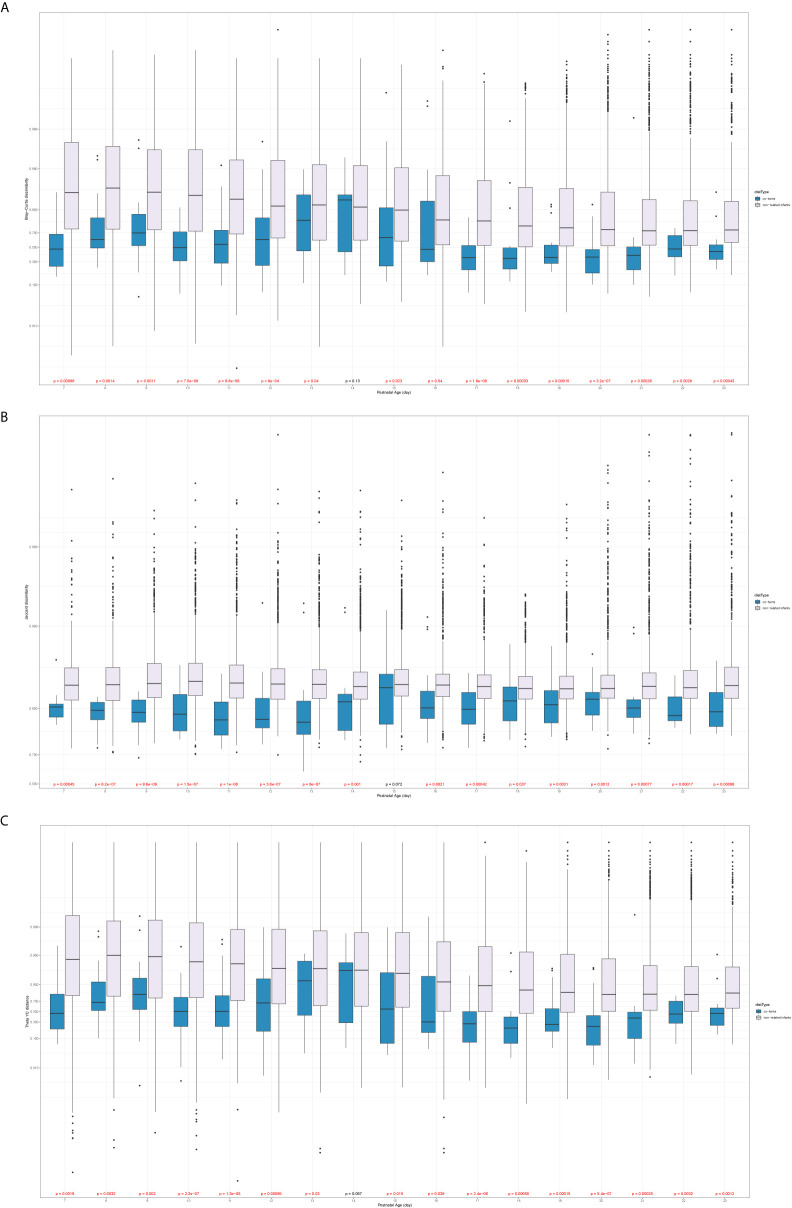
Comparison of the β-diversity of the co-twins/triples with non-related infants **from different sex** (**A**: Bray-Curtis dissimilarity; **B**: Jaccard dissimilarity; **C**: Theta YC distance). x-axis: the averaged postnatal age for sample pairs; y-axis: the logit-transformed β-diversity measures between 0 and 1; p values: Mann-Whitney U test, colored in red if it is significant.

The sample pairs were considered in this comparison if and only if they had different gender. The co-twins/triplets pairs with the *different sex* can only come from the Mixed T/T group, while the non-related infants’ pairs could be constructed within the Mixed T/T, across Female T/T & Male T, and among all three twin types groups. There was no significant difference among the three constructions of non-related infant pairs by the Mann-Whitney test. Thus, the non-related infants’ pairs could be assumed homogenous, and any non-related infants’ pairs satisfied with different sex were included to increase power. **Figure 4** presents the dissimilarity index between sample pairs of *different sex* against their averaged postnatal age. For all three types of dissimilarities, the β-diversities of non-related infants were statistically greater than that of the co-twins/triplets from day 7 to day 23 of their hospital stay. Moreover, the differences in the β-diversity between the non-related infants and co-twin infants decreased over time.

### Compositions of Gut Microbiota Between Male and Female Infants

**Figure 5:**
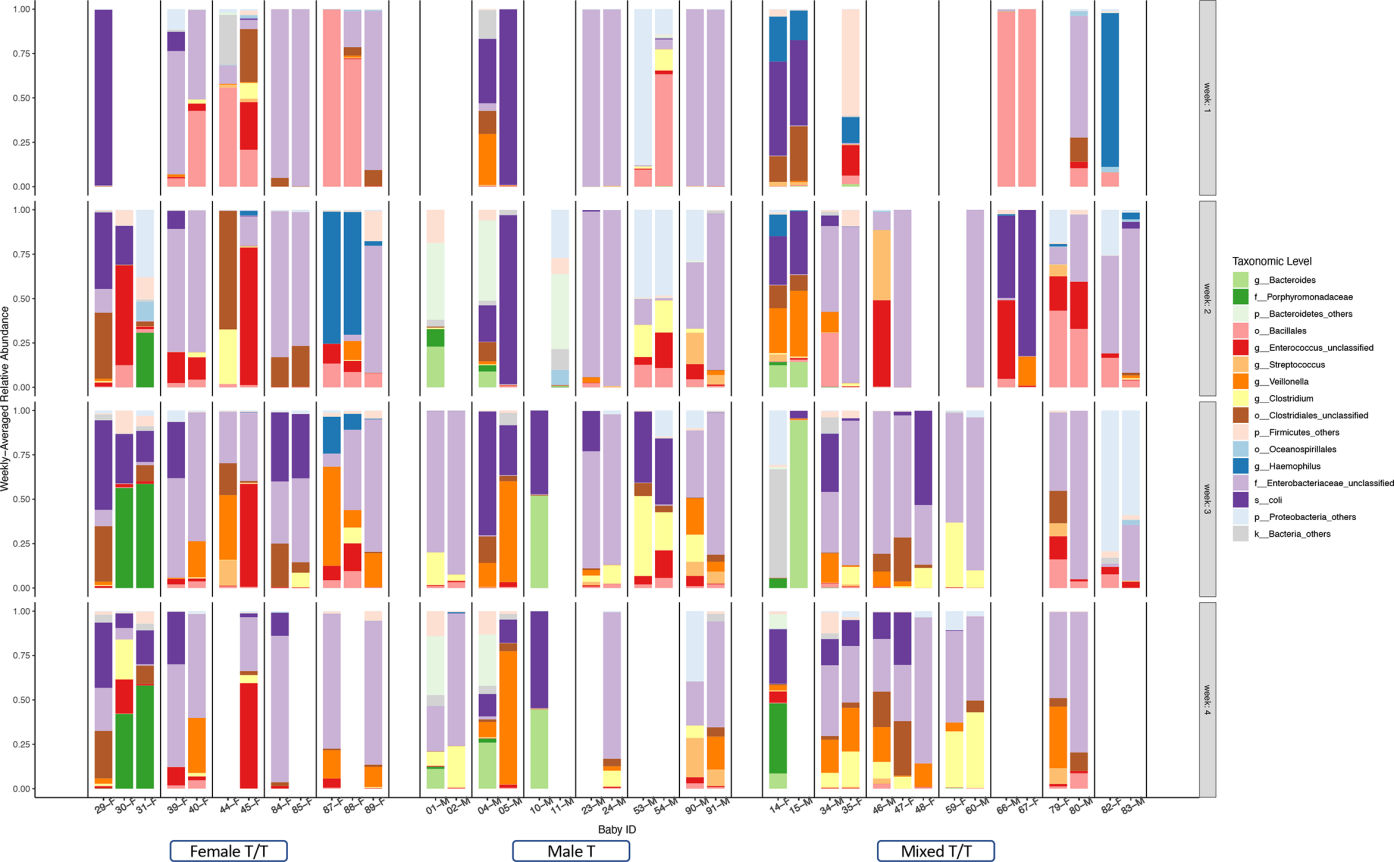
Summarized weekly relative abundance of taxonomic levels for each infant among Twins/Triplets. The relative abundance of the included twins/triplets was presented on a weekly basis in the first four weeks of NICU stay. Each column presents one infant, the sex and study-ID of each infant was listed on the bottom. The twins/triplets from the same mother were juxtaposed and babies from different mothers were separated by a black dotted line. Blank columns indicate no stool sample was obtained in that week. Different colors of the bar represent selected taxonomic levels.

The weekly relative abundance of taxonomic levels for each infant was summarized according to the dominating difference between Female T/T, Male T, and Mixed T/T (**Figure 5**). **Figure 5** shows the related abundance of each baby in each week of the NICU stay with each column representing samples from a baby (ID and sex in the bottom) and each row presenting the samples collected in a week. To compare and contrast the similarity of the abundance, we juxtaposed babies from the same mother together. Samples collected from the Female T/T and Male T were more similar compared with those in Mixed T/T. For example, Female twins’ pairs 39-F and 40-F (fourth and fifth column) had a similar abundance of Enterococcus unclassified and Enterobacteriaceae unclassified almost every week (row 1-4). The abundances in babies from different twins’ pairs are different such as different abundance between female babies 31-F and 39-F, the difference between female babies 40-F and 44-F. Male twins’ pairs also had similar abundances such as pairs 04-M and 05-M, pairs 23-M and 24-M. Mixed twins/triplets from the same mother also had similar abundances such as pair 14-F and 15-M, 77-F and 78-M.

**Figure 6:**
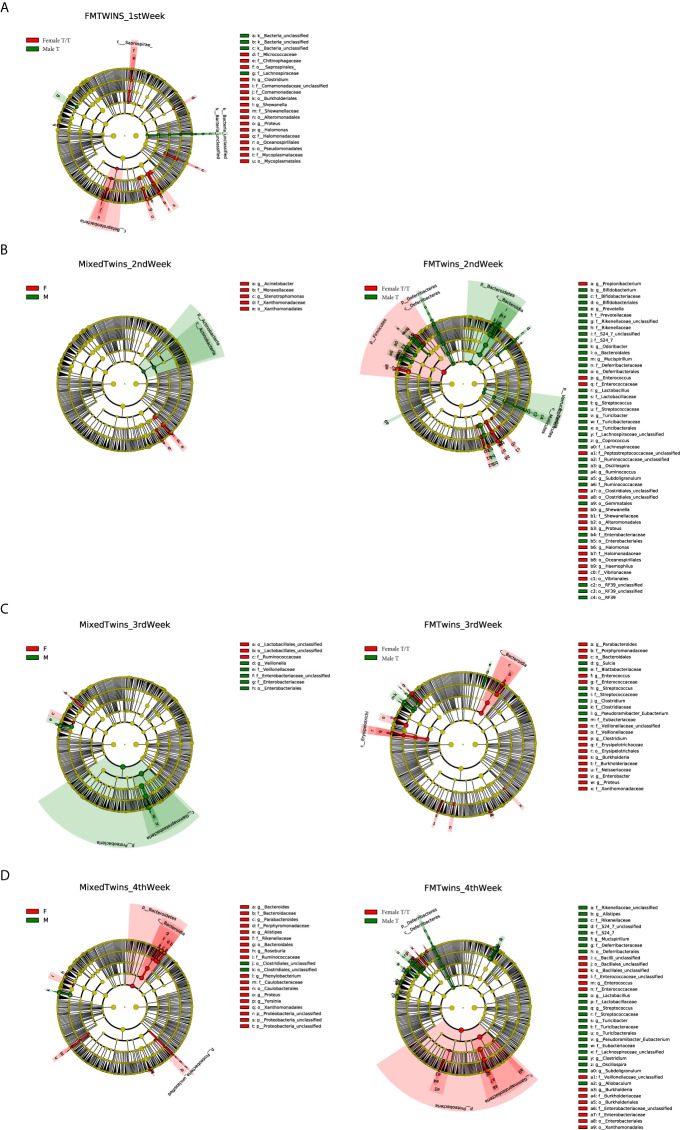
Compositional difference of gut microbiota between Twins/Triplets. The alpha value for the factorial Kruskal–Wallis test was set to 0.05 as well as for the pairwise Wilcoxon test, and the threshold on the logarithmic LDA score for discriminative features was set as 2.0. **(A–D)** show different compositions between male and female infants in the 1st, 2nd, 3rd, and 4th week of NICU stay, respectively. **(A)**. The compositional difference of gut microbiota between Female T/T and Male T in the 1st week of NICU stay. **(B)** Difference between female and male co-twins of the Mixed T/T pairs (left); difference between Female T/T and Male T in the 2nd week of NICU stay (right). **(C)** Difference between female and male co-twins of the Mixed T/T pairs (left); difference between Female T/T and Male T in the 3rd week of NICU stay (right). **(D)** Difference between female and male co-twins of the Mixed T/T pairs (left); difference between Female T/T and Male T in the 4th week of NICU stay (right).

Compositional differences of gut microbiota between the two sexes were also compared using Linear discriminant analysis Effect Size (LEfSe). We explored the weekly difference between the two sexes within co-twins of the Mixed T/T group as well as the sex differences of microbiome compositions in the Female T/T and Male T groups. **Figures 6A–D** show the different compositions between male and female infants in the 1^st^, 2^nd^, 3^rd,^ and 4^th^ week of NICU stay, respectively. Only Female T/T and Male T had significant abundant features in the first week (**Figure 6A**), no significant difference was found between co-twins from the Mixed T/T group in the first week. The difference between the two sexes within co-twins of the Mixed T/T was significant during the second, third, and fourth week of the NICU stay (**Figures 6B–D**). The difference between females and males among Mixed T/T was also significant in the second, third, and fourth week of the NICU stay (**Figures 6B–D**).

The Female T/T showed a higher abundance of *Enterococcaceae* in the 2^nd^, 3^rd,^ and 4^th^ week. The Male T had a higher abundance of *Lactobacillaceae* and *Streptococcaceae* in the 2^nd^ week, *Clostridiaceae* and *Streptococcaceae* in the 3^rd^ week, and *Lactobacillaceae*, *Eubacteriaceae*, and *Streptococcaceae* in the 4^th^ week. The numbers of significant abundant features between the Female T/T and Male T groups were much larger than those in females and males within the co-twins of the Mixed T/T group in the 2^nd^, 3^rd^, and 4^th^ week (180 *vs* 18, 85 *vs* 19, 154 *vs* 46), showing that the sex differences of gut microbiota composition in non-related infants (differences between Female T/T and male T) were greater than those in co-twin pairs (sex difference in Mixed T/T).

### Metabolic Profiles of Gut Microbiota Between Male and Female Infants

**Figure 7:**
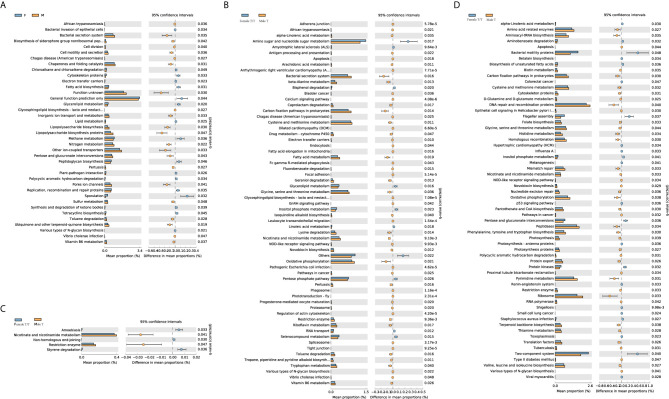
Different metabolic profiles of gut microbiota between Twins/Triplets. Kruskal-Wallis test was used to compare the difference of the KEGG pathway (level 3). Benjamini-Hochberg procedure was used to decreases the false discovery rate for multiple test corrections. The p-value was set as 0.05. **(A)** KEGG pathway difference between female and male co-twins of the Mixed T/T pairs during the 3^rd^ week of NICU stay. **(B)** KEGG pathway difference between Female T/T and Male T during the 2^nd^ week of NICU stay. **(C)** KEGG pathway difference between Female T/T and Male T during the 3^rd^ week of NICU stay. **(D)** KEGG pathway difference between Female T/T and Male T during the 4^th^ week of NICU stay.

The different metabolic profiles of gut microbiota between the two sexes were explored using STAMP. The weekly differences of the predicted metabolic profiles in level 3 of the KEGG pathway category were compared between female and male co-twins of the Mixed T/T pairs, as well as between the Female T/T and Male T groups. Female and male co-twins of the Mixed T/T pairs only had significant differences during the 3^rd^ week (**Figure 7A**), no significant difference was found in the 1^st^, 2^nd^, and 4^th^ week of NICU stay. However, significant differences were observed between Female T/T and Male T groups in the 2^nd^, 3^rd^, and 4^th^ week (**Figures 7B–D**), but not in the 1^st^ week. Males tended to have more abundant metabolic profiles in level 3 of the KEGG pathway category than females. For example, Male T groups tended to have more abundant metabolic profiles related to immune response and inflammatory mediation, e.g., “Nicotinate and nicotinamide metabolism” and “Tryptophan metabolism”.

## Discussion

The present study discovered the temporal and dynamic evolution of gut microbiota and sex-specific microbial features among preterm twins/triplets during their first 28 days of life at the NICU. Here, our study longitudinally identified the gut microbial communities of 39 preterm infants (3 twins and 2 triplets of Female T/T; 6 pairs of Male T; and 6 twins and 1 triplet of Mixed T/T). We found that microbial richness and diversity increased in all twins/triplets along with increasing postnatal days, as well as infants of co-twins/triplets shared more similar features than un-related infants from different twin pairs. Importantly, our study is one of the first that addressed the sex differences in microbiome development in twin pairs during their early life. Results showed that Female T/T and Male T groups were significantly different in gut microbiome diversity, compositions, as well as predicted metabolic profiles, meanwhile, females and males were also significantly dissimilar within their co-twin/triplet pairs of the Mixed T/T group. The Female T/T, Male T, Mixed T/T had significantly different variations among a-diversities (**Figure 1**). In addition, females tended to have greater variations in a-diversity (Sobs and Shannon) than males within Mixed T/T (**Figure 1**).

Preterm infants had more similar gut microbiome diversity (**Figures 2**–**4**) and compositions (**Figure 6**) in co-twins/triplets than between unrelated infants in our study. This result is consistent with previous studies showing that related preterm twins shared more similar microbiota compared to the unrelated infants, reflecting the host genetic and immune-regulatory influence on the microbiome development, even in the complex NICU environment (Stewart et al., 2013; Lim et al., 2015; Hill et al., 2017). Another explanation could be that shared utero environment and similar NICU experiences of the twins/triplets are responsible for the more similar microbiome profiles within the pairs. Fewer studies have investigated the gut microbiome of preterm twins in NICU, but one study showed that twins shared more microbes than non-twin pairs at their 5 months age to 5 years old (Zhou et al., 2016). One study compared the septic preterm twins with their non-septic twin controls and found that dysbiosis of gut microbiota presented in the septic twin infants, with a predominance of *Enterobacteri*a and reduction of *Bacteroides* and *Bifidobacterium spp* (Cernada et al., 2016). Notably, subjects included in this study were healthy preterm infants with no major health concerns. Microbiome data obtained in this study may not be generalized to twins with critical health issues. Further studies could also confirm the different variations of diversities among Female T/T and Male T/T, as well as among females and males among Mixed T/T.

Infant PNA and mother’s breast milk feeding were found to be the primary drivers to increase the gut microbiome richness and diversity in preterm infants of our study. Even though co-twins shared more similar gut microbiome profiles than among the non-related infants after birth, the microbial diversity patterns showed that the dissimilarity gradually decreased among the non-related twins but increased among co-twin infants from week 1 to 4 (**Figures 2** **–** **4**), which may reflect the environmental impact on the gut microbiome development. As one of the most important environmental factors, feeding significantly affected the development of infant gut microbiome, by which more mother’s breast milk intake was associated with increased gut microbial diversities (**Table 3**). In the current study, Female T/T received less mothers’ breast milk in the first week (**Table 2**), but diversity among females appeared higher, suggesting that there may other interactive effects of sex (as female) and other environmental factors on the diversity of preterm infants (Cong et al., 2016b). Even other studies (Bezirtzoglou et al., 2011; Lee et al., 2015) reported that formula feeding increased the diversity of gut microbiota among infants, the small sample size of and different ethnicities of included neonates should be addressed. Our results are consistent with previous studies (Cong et al., 2016a; Cong et al., 2017; Cai et al., 2019; Chen et al., 2020; Xu et al., 2020) and demonstrate that antioxidant and anti-inflammatory factors contained in the raw breast milk may protect the gut microbiome establishment and mitigate dysbiosis in preterm infants (Ramani et al., 2018; Hård et al., 2019; Chen et al., 2020; Wang et al., 2020). No significant difference of Shannon diversity index and Sobs were found between different twins/triplets types, this may be compromised by the significant different gestational age between different twins/triplets types since gestational age was reported to significantly influence Shannon diversity index and Sobs among preterm infants (La Rosa et al., 2014).

Most interestingly, our study showed significant differences in gut microbial diversity and compositions between the male and female infants in accordance with the differences between the Female T/T and Male T groups, as well as the dissimilarity between females and males in the Mixed T/T group (**Figures 1**, **6**). Our analysis and comparison of same-sex twins also demonstrate that sex impacts colonization due to environmental factors as well as the microbial development as shown in **Figures 3**, **6**. Females tended to have a higher microbial diversity than males and this finding may shed light on the underlying mechanisms of male disadvantage or female advantage in perinatal complications, and health and neurodevelopmental outcomes (Cong et al., 2016a). For instance, a study reported that male infants in mixed-sex twin pairs have significantly higher mortality and morbidity compared with their female siblings (Zhao et al., 2017). Another study showed that female preterm infants equipped with more mature suck, swallow, and breathe skills, and initiated independent oral feeding one day earlier compared with male counterparts (Van Nostrand et al., 2015). The mechanism of sex differences in infant health is still under-discovered. The sex-specific hormonal, genetic, and immunological attributes may contribute to the different organ maturation as well as gut microbiome development between sexes, thus leading to male disadvantage or female advantage of preterm outcomes (Jašarević et al., 2016). It should be noted that the environment could sharply shape the preterm infants’ gut microbiota (Rinninella et al., 2019; Selma-Royo et al., 2020).

The LEfSe results in our study indicated that females and males had significantly different gut microbiota compositions over the first four weeks of early life, with *Enterococcaceae* dominated in females and *Streptococcaceae* dominated in males. In the analysis of predicted metabolic profiles, we further found that males tended to have more abundant metabolic profiles related to the immune response and inflammatory mediation, such as the nicotinate and nicotinamide metabolism pathway (**Figure 7**). These pathways play pivotal roles in regulating cellular processes of oxidative stress and immune activation, energy metabolism, and biosynthesis, and also involved in degenerative disorders and aging (Orlandi et al., 2020). These differences may relate to the sex differences in the hypothalamic-pituitary-adrenal (HPA) axis’ response to early life stress among preterm infants, mediated by HPA produced glucocorticoids, cortisol, and other hormones (Cong et al., 2015; Jena et al., 2020). Limited studies are discovering gut microbiome metabolism in human infants. An animal study showed that the nicotinate and nicotinamide metabolism was one of the key relevant metabolic pathways involved in weaning stress-induced gut microbiota dysbiosis in piglets (Li et al., 2018). Adult study (Guo et al., 2020) reported that the abundance of *Enterococcaceae* was related to the function of promoting hematopoiesis and attenuating gastrointestinal damage, which was dominated in female infants in our study, and the abundance of *Streptococcaceae* was related to radiation-induced gut damage among adults with cancer, which was dominated in male infants in our study. Therefore, the male disadvantage in premature health outcomes may be mediated by the gut microbiome profile and metabolisms through the host-microbiome metabolites interactions (Xiong et al., 2017). Further studies are needed to focus on the potential sex-specific gut microbiome functions and their linkages with health outcomes in early life and across the lifespan.

Several limitations of this study should be addressed. Preterm twins/triplets from this study were only recruited in two medical centers in Northeastern of the United States. Preterm twins/triplets in other settings may experience different NICU care and feeding regimens that may contribute to various trajectories of microbial development. Also, the preterm twins/triplets included in this study received less antibiotics such as ten infants did not receive any antibiotic in the NICU. Maternal data of antibiotics use were not collected in the parent study. Therefore, the regression analysis didn’t incorporate the antibiotic data due to the limited sample size of infants who had antibiotic use after the first three days during the NICU stay, which might influence the microbiomes community. Another limitation is that only feeding frequency instead of the detailed feeding regimes such as the composition of and the volume of the formula was recorded in this study. The aforementioned issues may impede the generalization of this study.

The gut microbiome influences many significant aspects of human health and behavior, especially in early life. Our twin study showed that sex has a significant impact on gut microbiome diversity, compositions, and predicted functions. The PNA, feeding, and Apgar score also significantly affect the Shannon index indicating genetic and environmental interaction on gut microbiome development. Our novel findings of sex-specific gut microbial diversity, component, and metabolism pathway “Nicotinate and nicotinamide metabolism” and “Tryptophan metabolism” may provide mechanistic insight into sex differences in infant outcomes and disease prevalence, such as different preterm mortalities between females and males, as well as different preterm mortalities between female and their related male pairs within mixed twins/triplets. Further studies could explore the contribution of specific species on preterm outcomes such as neurodevelopment disparities between female twins/triplets and male twins/triplets, between female and their male pairs within mixed twins/triplets. Our findings by using 16S rRNA sequencing data should also be validated by analysis of shotgun metagenomics sequencing data since the metabolic pathways were predicted in this study. Sex and environmental factors such as feeding and length of NICU stay should be considered in designing future research and in developing personalized interventions for vulnerable high-risk infants.

## Data Availability Statement

The raw data supporting the conclusions of this article will be made available by the authors, without undue reservation.

## Ethics Statement

The studies involving human participants were reviewed and approved by University of Connecticut and Connecticut Children’s Medical Center. Written informed consent to participate in this study was provided by the participants’ legal guardian/next of kin.

## Author Contributions

XC and JC contributed to the conception or design of the research. XC, JC, HL, SH, M-HC, WX, and KM contributed to the acquisition, analysis, or interpretation of the data and drafted the manuscript. All authors contributed to the article and approved the submitted version.

## Funding

This publication was supported by the National Institute of Nursing Research of the National Institutes of Health (NIH-NINR) under Award Number K23NR014674 and R01NR016928, and Affinity Research Collaboratives award through the University of Connecticut Institute for Systems Genomics.

## Conflict of Interest

The authors declare that the research was conducted in the absence of any commercial or financial relationships that could be construed as a potential conflict of interest.

## Publisher’s Note

All claims expressed in this article are solely those of the authors and do not necessarily represent those of their affiliated organizations, or those of the publisher, the editors and the reviewers. Any product that may be evaluated in this article, or claim that may be made by its manufacturer, is not guaranteed or endorsed by the publisher.
