# Supplementary figures and images for: Sex Differences in Gut Microbial Development of Preterm Infant Twins in Early Life: A Longitudinal Analysis
Source: Front Cell Infect Microbiol. 2021 Aug 12;11:671074. doi: 10.3389/fcimb.2021.671074 (PMC8387566; doi:10.3389/fcimb.2021.671074)

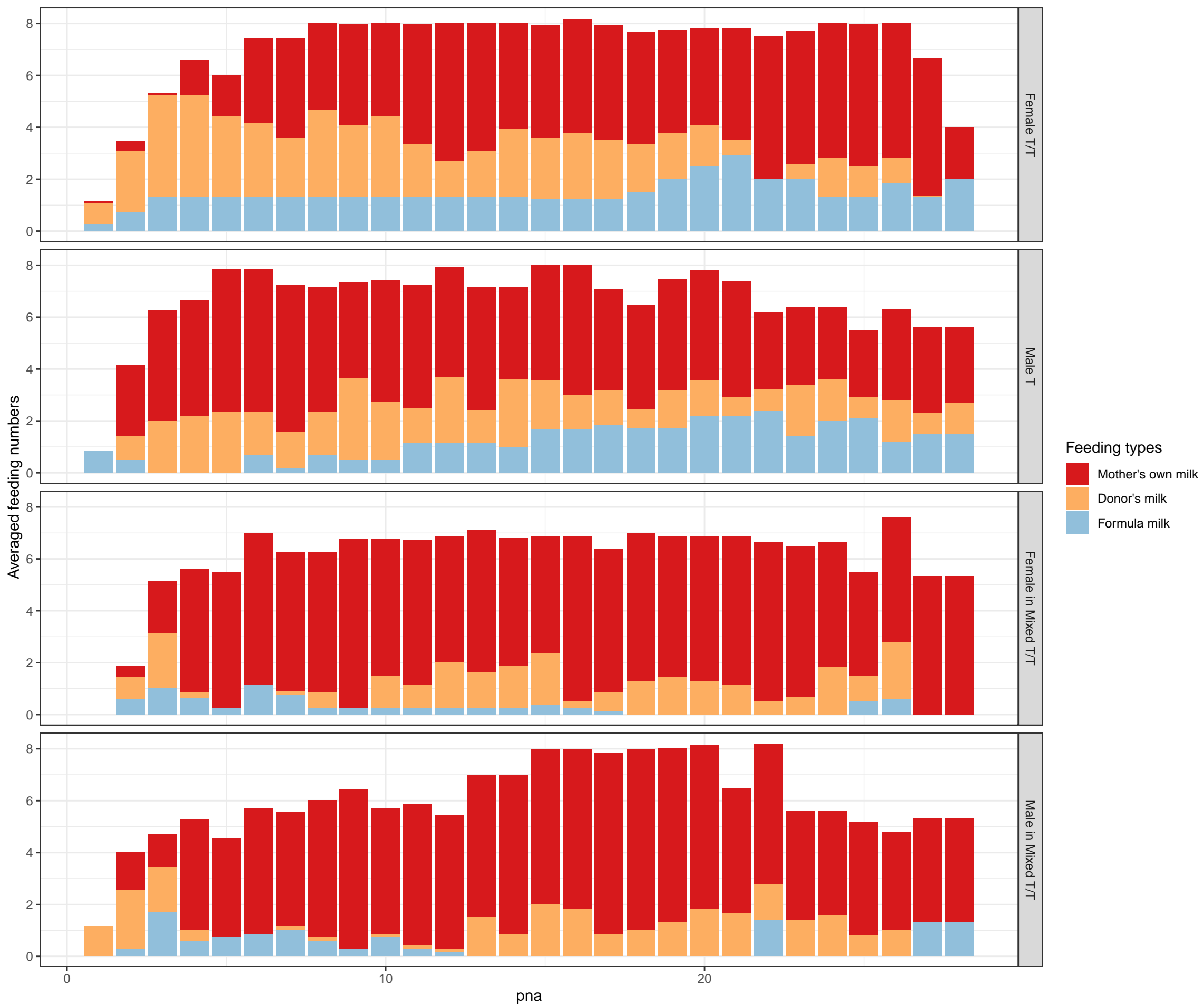

Supplement: Supplementary file 1 [file Image_1.pdf]
