# Supplementary material for: Sex Differences in Gut Microbial Development of Preterm Infant Twins in Early Life: A Longitudinal Analysis
Source: Front Cell Infect Microbiol. 2021 Aug 12;11:671074. doi: 10.3389/fcimb.2021.671074 (PMC8387566; doi:10.3389/fcimb.2021.671074)
Supplement: Supplementary file 2 [file Image_2.pdf]

S Figure 2 Flow chart of microbiota data analysis in this study

Step 1: Raw sequence data processing

The raw sequence data were processed following MARS Kendra's mothur batch file.

https://github.com/krmaas/bioinformatics/blob/master/mothur.batch

Bioinformatics Pipeline: mothur

The raw sequence data were achieved in NCBI (https://submit.ncbi.nlm.nih.gov/subs/sra/SUB8904718/).

Step 2: a-diversity description and comparison

General description of the α-diversity, including the observation of species (sobs) and Shannon diversity (Fig 1, generated by ggplot 2)

Factors associated with α-diversity, including sobs and Shannon diversity (Table 3: Linear Mixed Effect model by R)

Step 3: β -diversity comparison

Trend of β-diversity across three twin types, including Jaccard, and Theta YC dissimilarities index (Fig 2, generated by ggplot 2)

Comparison of the β-diversity of the co-twins/triples with non-related infants of the same sex, including the Bray-Curtis, Jaccard, and Theta YC dissimilarities index (Fig3, generated by ggplot 2)

Comparison of the co-twins/triples with non-related infants from the different sex, including the Bray-Curtis, Jaccard, and Theta YC dissimilarities index (Fig 4, generated by ggplot 2)

Statistical methods: Mann-Whitney U test by R

Step 4: Composition description and comparison

The weekly-averaged relative abundance of taxonomic levels for each infant (Fig 5, generated by ggplot 2)

Compositions of gut microbiota between male and female infants, including weekly difference between the two sexes within co-twins of the Mixed T/T group, and the sex differences of microbiome compositions in the Female T/T and Male T groups (Fig 6, generated by ggplot 2)

Bioinformatics Pipeline and Statistical methods: LEfSe (Segata, 2011) and Kruskal–Wallis test

Step 5: Metabolic profiles of gut microbiota between male and female infants

Metabolic profiles of gut microbiota between male and female infants, including weekly difference between the two sexes within co-twins of the Mixed T/T group, and the sex differences of microbiome compositions in the Female T/T and Male T groups

Statistical methods/Bioinformatics Pipeline: PICRUSt (Langille, 2013), STAMP (Parks, 2010), and Kruskal–Wallis test

Note: LEfSe, Linear discriminant analysis (LDA) effect size; PICRUSt, Phylogenetic Investigation of Communities by Reconstruction of Unobserved States; STAMP, Statistical Analysis of Metagenomic Profiles

References:

Langille, M. G. I., Zaneveld, J., Caporaso, J. G., McDonald, D., Knights, D., Reyes, J. A., et al. (2013). Predictive functional profiling of microbial communities using 16S rRNA marker gene sequences. *Nat. Biotechnol.* 31, 814–21. doi:10.1038/nbt.2676.

Parks, D. H., and Beiko, R. G. (2010). Identifying biologically relevant differences between metagenomic communities. *Bioinformatics* 26, 715–721. doi:10.1093/bioinformatics/btq041.

Segata, N., Izard, J., Waldron, L., Gevers, D., Miropolsky, L., Garrett, W. S., et al. (2011). Metagenomic biomarker discovery and explanation. *Genome Biol.* 12, R60. doi:10.1186/gb-2011-12-6-r60.
